# Synchronous Fluorescence as a Sensor of Trace Amounts of Polycyclic Aromatic Hydrocarbons

**DOI:** 10.3390/s24123800

**Published:** 2024-06-12

**Authors:** Suresh Sunuwar, Andrew Haddad, Ashlyn Acheson, Carlos E. Manzanares

**Affiliations:** Department of Chemistry & Biochemistry, Baylor University, 101 Bagby Avenue, Baylor Sciences Building E-216, Waco, TX 76706, USA; suresh_sunuwar1@baylor.edu (S.S.); andrew_haddad1@baylor.edu (A.H.); ashlyn_acheson1@baylor.edu (A.A.)

**Keywords:** fluorescence, synchronous, aromatic hydrocarbons, detection limit, astrochemistry

## Abstract

Synchronous fluorescence spectroscopy (SFS) is a technique that involves the simultaneous detection of fluorescence excitation and emission at a constant wavelength difference. The spectrum yields bands that are narrower and less complex than the original excitation and emission bands. The SFS bands correspond uniquely to the fluorescing molecule. Our investigation focuses on evaluating the sensitivity of the SFS technique for the detection and quantitation of PAHs relevant to astrochemistry. Results are presented for naphthalene, anthracene, and pyrene in three different solvents: n-hexane, water, and ethanol. SF bands are obtained with a constant wavelength difference between the peak excitation and emission wavelength (Δλ = λ_ex_ − λ_em_) at a concentration ranging from 10^−4^ to 10^−10^ M. Limit of detection (LOD) and limit of quantitation (LOQ) calculations are based on integrated SF band areas at different concentrations. Spectra of 23 pg/g of anthracene, 16 pg/g, and 2.6 pg/g of pyrene are recorded using ethanol as the solvent. The PAHs exhibit detection limits in the fractions of parts-per-billion (ng/g) range. Through comparison with similar prior studies employing fluorescence emission, our findings reveal a better detectability limit, demonstrating the effectiveness and applicability of the SFS technique.

## 1. Introduction

Synchronous fluorescence spectroscopy (SFS) is a powerful analytical technique that allows for the simultaneous detection of both excitation and emission wavelengths at a constant wavelength difference, producing an SF band with characteristic peaks [1]. This unique approach provides an effective means to analyze the fluorescence properties of a sample. The SFS technique developed by Lloyd [2] has found extensive applications, particularly in the detection of PAHs [3,4,5]. The direct detection of PAHs in astrochemical environments remains challenging due to their low concentration and the instrument’s limitations for detection. PAHs, much like on Earth, are also prevalent in diverse astrophysical environments, including molecular clouds [6,7], interstellar grains [8] and dust particles [9,10], planetary surfaces and atmospheres [11,12,13,14,15,16], cometary surfaces [17], meteorites [18,19,20], and asteroids [21]. They are of significant interest due to their potential role in astrochemical processes. While techniques such as gas chromatography [11], mass spectrometry [12,17], and fluorescence spectroscopy [22,23,24] have been employed for astrochemical PAH detection, the use of SFS remains largely unexplored. The direct application of SFS in this context presents a novel avenue for the investigation of extraterrestrial samples.

Anthracene, naphthalene, and pyrene were selected for their significance in astrochemistry [25,26,27,28,29]. These molecules, representing the simplest two-, three-, and four-ringed PAHs, allow for a comprehensive examination of a broad class of aromatic compounds with varying molecular weights. The choice of n-hexane and water as solvents was guided by their astrochemical relevance, considering that water ice is a dominant species in dense interstellar clouds [30], and water and n-hexane ices are significant components found in cometary nuclei as matrix mixtures alongside PAH compounds [30,31,32,33].

The spectra of ethanol and the simple sugar glycolaldehyde have been detected in a comet (Lovejoy) [34]. In our solar system, the icy surface of Jupiter’s moon, Europa, is thought to lie on top of a global ocean. Signatures in some Hubble Space Telescope images have been associated with assumed water plumes rising above Europa’s surface, providing support for the ocean theory [35]. On Saturn’s moon Enceladus, a south polar vapor plume, as observed by the Cassini Ion and Neutral Mass Spectrometer (INMS), is composed of H_2_O (~90%) with <1% CO_2_, CH_4_, NH_3_, H_2_, and trace organics, and has been linked to a salty subsurface ocean [36]. Water ice has been found in the polar regions of Mars [37]. The lakes of Saturn’s moon Titan have non-polar hydrocarbons (methane, ethane, and propane) as their main components [38]. The spectra of PAHs in n-hexane should be similar to the spectra in smaller hydrocarbons.

The experiments presented in this research work aim to investigate the limit of detection (LOD) and limit of quantitation (LOQ) using synchronous fluorescence spectra of PAH compounds that hold astrochemical significance, namely anthracene, naphthalene, and pyrene in n-hexane, water, and ethanol solvents. Synchronous fluorescence bands are shown for concentrations between 10^−4^ M and 10^−9^ M, but calculations of LOD and LOQ are performed based on SF band areas with concentrations between 10^−8^ to 10^−10^ M, where the plots are linear. This study assesses the potential of SFS to detect trace organic compounds in astrochemical environments and proposes the SFS technique as a promising instrumental technique for space exploration considering the current need for more sensitive techniques that are non-intrusive for the accurate determination of organic compounds. This technique can also be applied to analyze trace amount of PAHs in water samples on Earth.

## 2. Materials and Methods

### 2.1. Materials

Anthracene of 98% purity and pyrene of 98% purity were purchased from Aldrich. Naphthalene of 99% purity was obtained from Mallinckrodt. ACS Certified-grade n-hexane of 98.5% purity was purchased from Fischer Scientific. Ethanol of 200 proof was obtained from Decon Laboratories. An in-house supply of deionized water from the laboratory was used. Initially, stock solutions of 1 × 10^−4^ M were prepared by mixing the appropriate amount of the sample with the required volume of solvents and stored in the dark. All working solutions were prepared from the stock solutions just prior to measurement. Shimadzu RF-6000 (Shimadzu Corporation, Kyoto, Japan) was the instrument of choice for all synchronous fluorescent measurements. For sample measurement, a 1 cm pathlength cuvette from FireflySci was used.

### 2.2. Methods

Synchronous fluorescence measurements were carried out in a model RF-6000 Spectrofluorometer from Shimadzu. The Δλ values for SF measurements were obtained from the difference between maximum peak emission and maximum peak excitation wavelengths. They were set for anthracene at Δλ = 44 nm in water, n-hexane, and ethanol, respectively. The fact that Δλ is the same irrespective of the solvent means that the solvent does not change the shape of the bands nor the separation of the maximum of the excitation and emission bands. A similar situation occurs for naphthalene with Δλ = 50 nm for the three solvents. The solvents show a strong effect in the synchronous spectra of pyrene, changing the separation between the maximum of the excitation and emission bands or Δλ = 40 nm, 50 nm, and 60 nm for pyrene in water, n-hexane, and ethanol, respectively. The scans were taken at a speed of 300 nm/min with 0.2 nm data intervals. The excitation and emission slit widths were set at 5 nm each. The final spectrum was obtained from an average of 20 scans. The data were exported in CSV format from the instrument to Microsoft Excel (version 2311). Regression analysis was conducted utilizing the LINEST regression analysis tool in Excel to obtain the linear regression parameters from the band area versus concentration (ng/g). The limit of detection is defined as $LOD=3.3\times \left(\frac{\sigma_{y}}{S}\right)$ and the limit of quantification as $LOQ=10\times \left(\frac{\sigma_{y}}{S}\right)$. In astrochemistry, it is common practice to report concentrations in terms of the mass of the solute/the mass of the solvent in ng/g. For very low concentrations, a good approximation is

(1) $$\left(\frac{ng\;(solute)}{g\;(solvent)}\right)=Molarity\times solute\;molar\;mass\times \frac{1}{solvent\;density\;}\times 10^{9}$$

The solvent density in (g/L) at room temperature is 659 for n-hexane, 789 for ethanol, and 1000 for water. The molar mass (g/mol) is 128.17 for naphthalene, 178.23 for anthracene, and 202.25 for pyrene.

## 3. Results and Discussion

### 3.1. Anthracene

**Figure 1:**
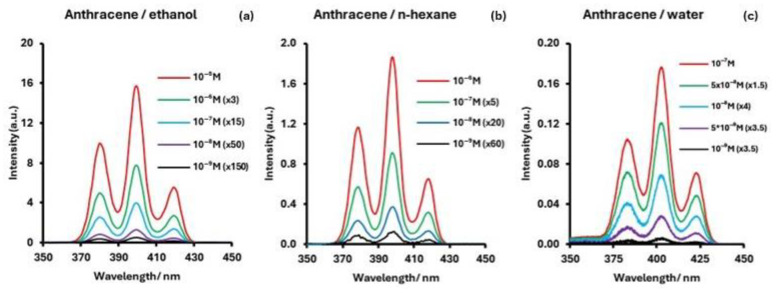
Synchronous fluorescence spectra (Δλ = 44 nm) of anthracene in (**a**) ethanol, (**b**) n-hexane, and (**c**) water. Intensities divided by 10^4^.

Anthracene has positively been identified in various celestial bodies, including the comet 1P/Halley [25], the Murchison meteorite [19], and sample extracts from the asteroid Ryugu [21]. Furthermore, it is also considered as a prime candidate for the origin of interstellar luminescence [26]. Hence, it is an important molecule contributing to our broader understanding of the chemical makeup and processes occurring in diverse cosmic environments. The SF spectra of anthracene in ethanol (a), n-hexane (b), and water (c) are shown in Figure 1. The intensities for all bands are divided by 10^4^.

**Figure 2:**
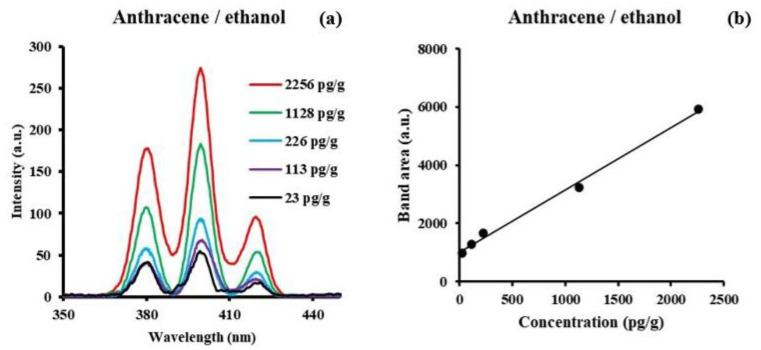
(**a**) Synchronous fluorescence spectra (Δλ = 44 nm) of anthracene in ethanol at trace level concentrations between 1 × 10^−8^ M (2.26 ng/g) and 1 × 10^−10^ M (22.6 pg/g). (**b**) Total band area versus concentration.

The SF spectra of anthracene in all three solvents consist of three bands, with the central band being the most intense. The peak fluorescence excitation and emission bands are very similar giving the same Δλ = 44 nm for all three solvents. The observed position of the band maxima are essentially the same or 399 nm in ethanol, 398 nm in n-hexane, and 403 nm in water. Figure 2 shows a representative example of anthracene in ethanol at low concentrations between 1 × 10^−8^ M (2.26 ng/g) and 1 × 10^−10^ M (22.6 pg/g).

The lowest concentration was obtained by averaging 100 signals with smoothing after the average. It is clear that concentrations in the order of picograms per gram (pg/g) are easily measured using this technique. The LOD values calculated for anthracene are 0.11 ng/g in n-hexane, 0.25 ng/g in ethanol, and 0.29 ng/g in water. The lowest LOD is for hexane with, a dipole moment of 0.09, followed by ethanol (1.66 D) and water (1.84 D). The LOQ values of anthracene are less than 1.00 ng/g in all solvents.

### 3.2. Naphthalene

**Figure 3:**
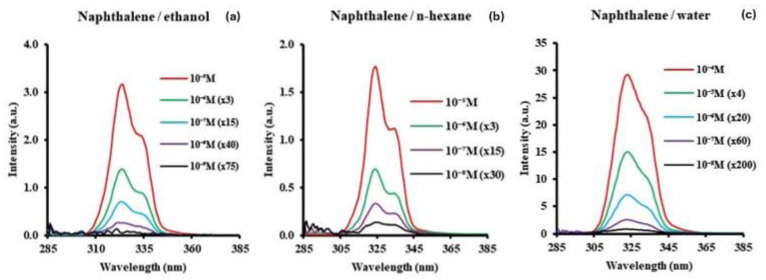
Synchronous fluorescence spectra (Δλ = 50 nm) of naphthalene in (**a**) ethanol, (**b**) n-hexane, and (**c**) water. Intensities divided by 10^4^.

Naphthalene, a molecule of astrochemical interest, has been observed in comets [17], and interplanetary dust particles [9]. Additionally, it has been detected in the interstellar medium in its cationic form [27]. Furthermore, naphthalene plays a pivotal role in key chemical pathways leading to the formation of higher-molecular-weight PAHs like anthracene in extraterrestrial environments [24]. Figure 3 shows the SF spectra of naphthalene in the three solvents.

**Figure 4:**
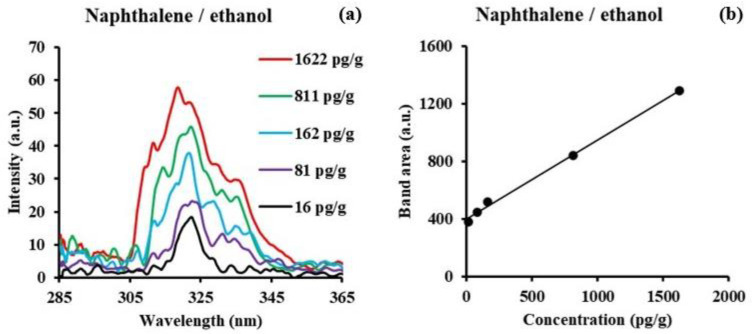
(**a**) Synchronous fluorescence spectra (Δλ = 50 nm) of naphthalene in ethanol at trace level concentrations between 1 × 10^−8^ M (1.62 ng/g) and 1 × 10^−10^ M (16.2 pg/g). (**b**) Total band area versus concentration.

The SF spectra of naphthalene in ethanol and n-hexane both consist of the peak maximum at 324 nm and a shoulder band at 336 nm. Since ∆λ was set at 50 nm in ethanol, hexane, and water, the consistency of the SF peak position is reasonable. The spectra in ethanol, n-hexane, and water are similar. The intensities for all bands are divided by 10^4^. Although there is little change in the shape of the SF spectra, the shoulder band in the spectrum in water seems less separated from the major band compared to those in the spectra in n-hexane and ethanol, where the shoulder bands are more resolved and distinctive. The overall concentration range was 10^−5^ to 10^−8^ M, except for naphthalene in ethanol. Figure 4a shows a representative example of naphthalene in ethanol at low concentrations between 1 × 10^−8^ M (1.62 ng/g) and 1 × 10^−10^ M (16.2 pg/g). The total integrated band of naphthalene in ethanol is shown in Figure 4b.

The lowest concentration was obtained by averaging 100 signals with smoothing after the average. Spectra for concentrations in the order of picograms per gram (pg/g) can be observed using this technique. The LOD values calculated for naphthalene are 0.14 ng/g in ethanol. Based on the results with ethanol, the LOD values of 4.42 ng/g in n-hexane and 3.52 ng/g in water could be lower, but concentrations below 10^−8^ M were not measured.

### 3.3. Pyrene

**Figure 5:**
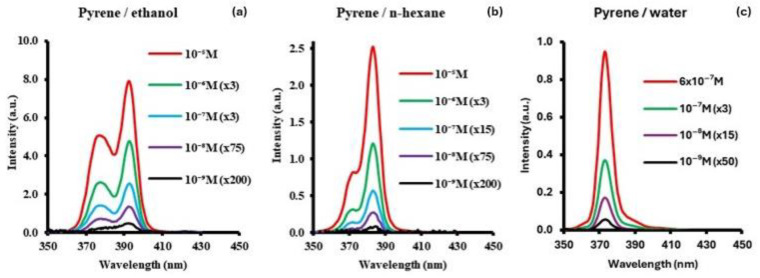
Synchronous fluorescence spectra (SFS) of pyrene in (**a**) ethanol at Δλ = 60 nm, (**b**) hexane at Δλ = 50 nm, and (**c**) water at Δλ = 40 nm. Intensities divided by 10^4^.

The tentative identification of pyrene has been performed in the comet P/Halley [28], the asteroid Ryugu [21], and has also been detected in the Murchison and Orgueil meteorites [10,19] in significant amounts. Additionally, it has also been detected in a stardust sample from the comet 81P/Wild [17]. Pyrene is an valuable component for PAH studies considering the fact that it is an important intermediate in the formation of large PAHs from smaller PAHs in the interstellar medium [29]. Figure 5 shows the synchronous fluorescence spectra of pyrene measured in three different solvents, namely ethanol (Figure 5a), n-hexane (Figure 5b), and water (Figure 5c), over a concentration range from 10^−5^ to 10^−9^ M. The intensities for all bands are divided by 10^4^.

**Figure 6:**
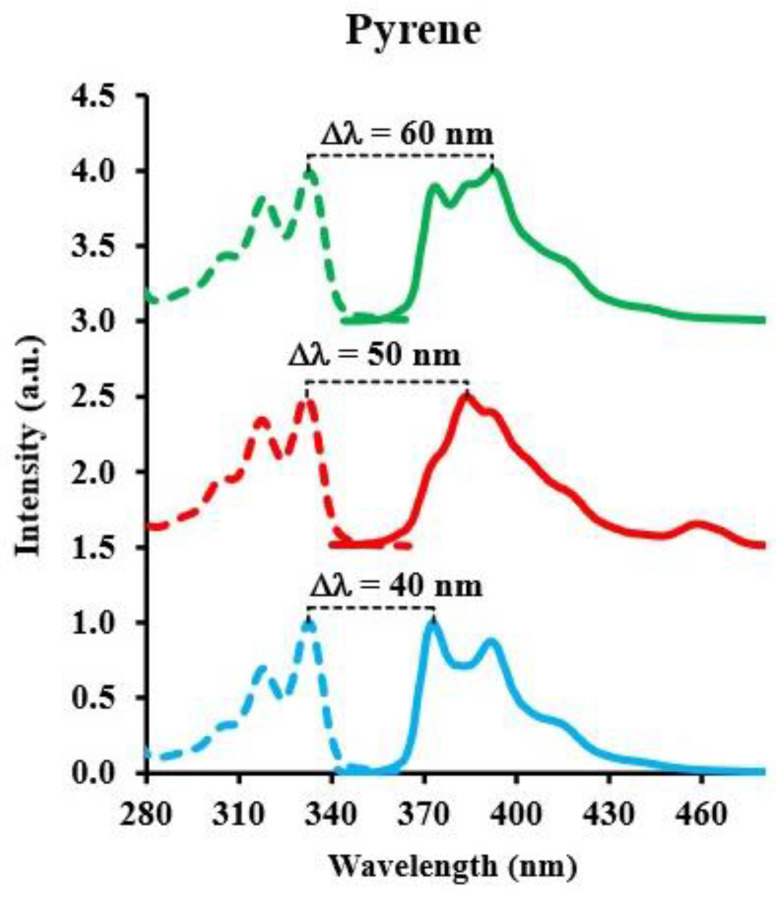
Solvent effect over the excitation and emission bands of pyrene in three different solvents. Green: excitation (dotted line) and emission (solid line) in ethanol. Red: excitation (dotted) and emission (solid) in n-hexane. Blue: excitation (dotted) and emission (solid) in water.

The SF spectra in ethanol consist of two bands: a broader, less intense band at 377 nm and a relatively narrower, more intense band at 393 nm. For the SF spectrum in n-hexane, a major band at 383 nm is observed, along with a less intense band towards a shorter wavelength at 373 nm. Unlike the SF spectra in n-hexane and ethanol, the spectrum in water consists of a single major band at 373 nm. Pyrene exhibits distinct peak emission wavelengths depending on the solvent used: 392.5 nm in ethanol, 383.0 nm in hexane, and 373.0 nm in water. The large differences in the emission peak wavelength are indicative of the influence of the solvent environment on pyrene emission. Figure 6 shows that while the excitation band peak position of pyrene remains the same in the three solvents, the emission band’s peak position and shape change.

**Figure 7:**
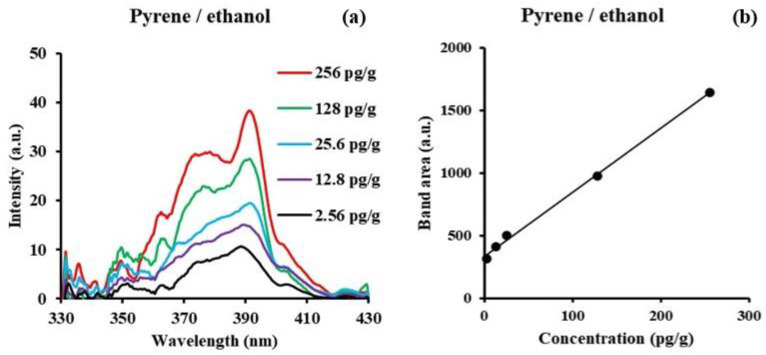
(**a**) Synchronous fluorescence spectra (Δλ = 60 nm) of pyrene in ethanol at trace level concentrations between 1 × 10^−8^ M (256 pg/g) and 1 × 10^−10^ M (2.56 pg/g). (**b**) Band area versus concentration.

Since the Δλ value in this work is the Stokes shift value (peak emission wavelength (λ_ex_)—peak excitation wavelength (λ_em_)), the solvent-influenced peak wavelength shift observed in the fluorescence spectrum can also be seen in the synchronous spectrum. The corresponding wavelength displacements (∆λ) for SF measurements were 60 nm, 50 nm, and 40 nm in ethanol, hexane and water, respectively. The red shifting of the SF peak from water to hexane to ethanol is consistent with the trend of increasing Δλ values being used. Additionally, the overall band width of the SF spectra in the three solvents correlate to the corresponding ∆λ used. The smaller the ∆λ value used, the narrower the bandwidth. The LOD values calculated for pyrene are 0.018 ng/g in ethanol, 0.44 ng/g in n-hexane, and 0.54 ng/g in water. Figure 7a shows a representative example of pyrene in ethanol at low concentrations between 1 × 10^−8^ M (2.56 ng/g) and 1 × 10^−10^ M (25.6 pg/g). The lowest concentration was obtained by averaging 100 signals with smoothing after the average. The total band area versus concentration is shown in Figure 7b. Concentrations in the order of picograms per gram (pg/g) can be measured using this technique.

## 4. Improvements in Synchronous Detection

Table 1 summarizes the results for anthracene, naphthalene, and pyrene in each of the solvents, water, ethanol, and hexane. The majority of limits of detection and quantitation are in fractions of parts per billion (ng/g). The LOD values obtained using a commercial instrument are of the order of magnitude found in astrochemical analysis.

For space exploration, a tunable pulse laser (instead of a lamp) is the indicated source for excitation and emission experiments, combined with a dispersion grating (instead of a monochromator). The combination of these instruments makes miniaturization and greater sensitivity possible. There are already methods in the literature that, although they are based on the detection of a single peak of fluorescence emission (instead of a complete band), have shown improved limits of detection in water. Richardson and Ando [39] determined the minimum detectable concentration for pyrene, naphthalene, and anthracene in water through the utilization of laser-induced fluorescence. The excitation laser was a Molectron UV 1000 N_2_ laser and a Molectron dye laser. This laser was usually operated at 30 Hz, which generally maximized the signal-to-noise ratio; typical peak powers of the doubled dye output were 2 to 5 kW, with approximately an 8 ns temporal bandwidth (FWHM). The minimum detectability was 0.5 pg/g for pyrene, 1.3 pg/g for naphthalene, and 4.4 pg/g for anthracene. Our values are higher because of the lamp instrument used. In this study, we point the way toward a detection method that is not only very sensitive but also can identify the compound based on the synchronous band, similar to the method applied with infrared absorption. The notable difference in the minimum detectable concentration can be attributed to the excitation sources employed. Richardson and Ando [39] used a laser, whereas our current experiment utilizes a xenon lamp. A laser, as an excitation source, is known for its higher photon densities and spatial coherence, contributing significantly to an more sensitive technique. In contrast, a xenon lamp, while serving as a valid alternative for some uses, lacks the photon density and coherence characteristics inherent in a laser. Schwarz and Wasik [40] also determined the limits of detection in water using a lamp and a photon counting method. The fluorescence excitation light was provided by a combination 60-watt deuterium lamp and an f/3.5 monochromator at a resolution of 3.2 to 6.4 nm. The limits of detection in water of pyrene, naphthalene, and anthracene for fluorescence emission detection of a single wavelength were found to be 0.15 ng/g, 0.03 ng/g, and 0.03 ng/g, respectively [40]. A distinguishing feature of the current work is the use of the synchronous fluorescence technique to determine the detection and quantitation limits of PAHs. This sets our study apart from previous works [39,40], where the conventional fluorescence emission technique was employed. Furthermore, our experiment incorporates additional solvents, namely ethanol and hexane.

This study provides insights into how solvents of different polarities and dielectric constants affect the sensitivity and detection capabilities of SFS. An approximation that has been applied to the energy difference between the absorption and emission of a fluorescent molecule in solvents without hydroxyl groups is described by the following equation [41]:

(2) $${\bar{\nu }}_{A}-{\bar{\nu }}_{F}=\frac{2}{hc}\left(\Delta f\right)\frac{{\left(\mu_{E}-\mu_{G}\right)}^{2}}{a^{3}}+constant$$

In this equation, *h* (= 6.6256 × 10^−27^ ergs) is Planck’s constant and *c* (= 2.9979 × 10^10^ cm/s) is the speed of light. The difference between the wavenumbers (cm^−1^) of the absorption and fluorescence emission (${\bar{\nu }}_{A}-{\bar{\nu }}_{F}$) is proportional to $\Delta f=\left(\frac{\varepsilon -1}{2\varepsilon +1}-\frac{n^{2}-1}{2n^{2}+1}\right)$, which involves the dielectric constant (ε) and refractive index (*n*) of the solvent, the difference in dipole moments of the excited state (E) and the ground state (G) of the fluorescent molecule ${\left(\mu_{E}-\mu_{G}\right)}^{2}$, and $a^{3}$, which is the volume of the spherical cavity where the molecule resides. Equation (2) is only an approximation, and has not been used for synchronous bands. By analogy with the difference in peak wavelength (or frequency) of the synchronous band, we can try to explain our results using some terms of the equation. The solvent participates in the equation through Δf and the dipole difference ${\left(\mu_{E}-\mu_{G}\right)}^{2}$. The dielectric constant and index of refraction of hexane (ε = 1.89, *n* = 1.37), ethanol (24.3, 1.35), and water (78.3, 1.33) provide Δf values of 0.001, 0.30, and 0.32 for n-hexane, ethanol, and water, respectively. The most important term in Equation (2) is the difference in dipole moments. Solute–solvent interactions with the formation of complexes change the symmetry of the molecule and the dipole moment in the excited state. For pyrene, the interaction with each solvent is very strong, to the point of forming complexes with them. In this case, a different vibronic level becomes important for each solvent and is responsible for the maximum of fluorescence emission. The wavelength separation for pyrene is in the order ethanol > hexane > water. Glushko et al. [42], found that the vibrational fine structure superimposed on pyrene monomer fluorescence is solvent-dependent at room temperature. The fine structure pattern of pyrene monomer fluorescence was found to be independent of the excitation conditions or collisional quenching, but highly dependent on solvent polarity. Variation in pyrene fluorescence’s fine structure was investigated in alkane and alkanol solvents and solvent mixtures. Although changes in the fine structure pattern were detected across the monomer fluorescence spectrum, the greatest difference occurred at the 373 nm and 384 nm peaks. In nonpolar environments, the 384 nm peak dominated the pyrene spectrum, whereas at a higher polarity, the 373 nm peak was the most intense. We found a similar result where the 384 nm emission peak of pyrene is dominant for n-hexane, while the 373 nm peak is dominant for water. In the case of ethanol, we found that although the 373 nm and the 384 nm peaks are present, there is another peak at 392 nm that produces the maximum emission.

Experimentally, we found that there are no appreciable differences between solvents for anthracene. The Δλ = 44 nm for anthracene with all of the solvents means that there is no strong interaction with any of the solvents. The same vibronic level is responsible for the maximum emission in all of the solvents. This could also be associated with the solvent-independent difference in dipole moment between the ground ($\mu_{G}$) and excited states ($\mu_{E}$). A similar explanation could be used for the behavior of naphthalene (Δλ = 50 nm) in the three solvents.

The SF results presented in this investigation show that this technique could be used as a sensor for fluorescent compounds. SF is as sensitive as liquid chromatography with fluorescence (LC/fluorescence) detection and gas chromatography/mass spectrometry (GC/MS). LC/fluorescence detection and GC/MS have been compared to determine PAHs in various samples [43]. The results indicate that both techniques generally yield comparable results for measuring PAHs in environmental samples (0.1 ng/g). As expected, LC/fluorescence is more suitable for accurately measuring molecules with a high fluorescence quantum yield like anthracene and perylene at low levels, while GC/MS provides more accurate results for compounds with a low fluorescence quantum yield. Both techniques require sample preparation. The Mars samples were analyzed with GC/MS [14], but with the disadvantage that pyrolysis of the sample was involved before the analysis, possibly destroying the original samples by inducing chemical reactions. In combination with a laser source, SF guarantees a sensitivity of detection at the pg/g level, with minimum sample preparation involved.

## 5. Conclusions

The unique capabilities of SFS, allowing the simultaneous scanning of excitation and emission wavelengths at a constant wavelength difference, have been employed to obtain the synchronous fluorescence spectra of PAH compounds, specifically pyrene, naphthalene, and anthracene in n-hexane, ethanol, and water. Spectra of parts per trillion (pg/g) concentrations of anthracene (23 pg/g), naphthalene (16 pg/g), and pyrene (2.6 pg/g) were recorded using ethanol as the solvent. This study explores the potential of synchronous fluorescence spectroscopy (SFS) as a novel and effective tool for the detection of PAHs with astrochemical significance. It can also be used for the detection of PAHs as contaminants in terrestrial water. The calculated limits of detection and quantitation in fractions of parts per billion show the potential of the SF technique for detecting trace fluorescent compounds. The results highlight synchronous fluorescence spectroscopy as a highly promising spectroscopic tool for the sensitive detection of trace amounts of PAHs.

## Figures and Tables

**Table 1:** Figures of merit for the synchronous fluorescence spectra of anthracene, naphthalene, and pyrene dissolved in ethanol, hexane, and water.

Molecule	Solvent	Concent. Range/M	SF Peak (nm)	Δλ/nm	LOD/ng/g	LOQ/ng/g
Anthracene	Ethanol	10^−8^–10^−10^	399.4	44	0.25	0.76
	Hexane	10^−7^–10^−9^	398.0	44	0.11	0.33
	Water	10^−7^–10^−9^	402.7	44	0.29	0.88
Naphthalene	Ethanol	10^−8^–10^−10^	324.0	50	0.14	0.42
	Hexane	10^−6^–10^−8^	324.0	50	4.42	13.39
	Water	10^−6^–10^−8^	323.0	50	3.52	10.65
Pyrene	Ethanol	10^−8^–10^−10^	392.5	60	0.018	0.055
	Hexane	10^−7^–10^−9^	383.0	50	0.44	1.33
	Water	10^−7^–10^−9^	373.0	40	0.54	1.62

Only data points in the linear region have been used to calculate LOD and LOQ.

## Data Availability

The data in this study are available upon request from the corresponding author.
